# eLabFTW as an Open Science tool to improve the quality and translation of preclinical research

**DOI:** 10.12688/f1000research.52157.2

**Published:** 2021-05-27

**Authors:** Michael Hewera, Daniel Hänggi, Björn Gerlach, Ulf Dietrich Kahlert

**Affiliations:** 1 Clinic for Neurosurgery, Medical Faculty, Heinrich Heine University Dusseldorf, Düsseldorf, NRW, 40225, Germany; 2 PAASP GmbH, Heidelberg, BW, 69118, Germany; 3 Beijing Neurosurgical Institute, Beijing, China

**Keywords:** Quality Management, Electronic Lab Notebook, ELN, Open Science, Reproducibility, Transparency

## Abstract

Reports of non-replicable research demand new methods of research data management. Electronic laboratory notebooks (ELNs) are suggested as tools to improve the documentation of research data and make them universally accessible. In a self-guided approach, we introduced the open-source ELN eLabFTW into our life-science lab group and, after using it for a while, think it is a useful tool to overcome hurdles in ELN introduction by providing a combination of properties making it suitable for small life-sceience labs, like ours. We set up our instance of eLabFTW, without any further programming needed. Our efforts to embrace open data approach by introducing an ELN fits well with other institutional organized ELN initiatives in academic research and our goals towards data quality management.

## Introduction

In the light of reports of non-replicable research results, new ways to strengthen an open science approach for better transparency, reproducibility and traceability are needed. ^1^ Reproducibility meaning, that following the proper formatted documentation, any other lab group with the necessary equipment and personnel should be able to obtain the same results of any experiment performed. ^2^ An easy way to increase the legibility, tidiness, and overall quality of documentation, can be the introduction of an electronic laboratory notebook (ELN) in a research unit. Publishing the ELN entries in an unaltered state together with the manuscript enhances open science by creating transparency and will ultimately support reproducibility. ^3^ Here, ELNs will help in the context of research data management to make the entire life cycle of data transparent and achieve better integration to the documentation process. ^4^ The pros and cons of using ELNs have been discussed for a long time. The main fields of discussion are usability, accessibility, costs, time effort and data safety. ^5^^,^^6^ In our experience, the use of an ELN has many benefits towards traditional paper-based notebooks, but it is often a challenge to convince researchers of the advantages of using an ELN. We believe that the use of eLabFTW, and open source software, is a particularly good approach to overcome this hurdle and ensure high research data quality in small, preclinical research groups like ours.

eLabFTW is a lightweight general-purpose ELN. It consists of an Experiment and a Database section, whereas the Experiment section is designed to create and edit experimental documentation and link it to entries of the Database section. These can be set by the administrator to be any type of object (Chemicals, cell lines, equipment, etc.). These two sections are complemented by a Teams section, in which equipment can be booked in a scheduler and experiment templates can be shared. Finally, eLabFTW provides an extensive search tool. ^7^

## Disadvantages and advantages of ELN on experimental and lab management level

Overall acceptance for the usage of ELNs in the academic field is rather low. This seems to be due to perceived disadvantages, when using ELNs. These include the costs of obtaining the software and to maintain the necessary infrastructure, the time needed to implement the ELN and adapt the lab habits, as well as the fear of data being stored in a cloud on a foreign server. ^6^ Another important point concerns the protection of personal data (e.g., patient information) from unauthorized access when working with primary samples from patients. ^6^ Other scientists fear that the software will be discontinued at one point in the future, making it necessary to search for a new ELN, with the uncertainty whether the data from the previous system can still be accessed and imported to the new system. ^6^ Furthermore, there is a constraint to the implemented features of the software, in contrast to the possibility to freehand layouts, graphs and text in traditional lab notebooks. ^8^ These perceived disadvantages could be hurdles for people who want to introduce ELNs in lab groups. We believe that the advantages of ELNs outweigh the listed disadvantages by far. These advantages lay especially in the fields of open science and reproducibility, data safety, availability, findability, as well as transparency and standardization of procedures. Additionally, to those most important factors, there are other advantages in using ELNs: Digital recording of research data makes it much easier for researchers, to find their older data as well as data of others. In addition, the sharing of data is simplified, which makes discussing, cooperation and troubleshooting of experimental procedures much easier. ELNs contain time saving mechanisms, such as automatic import of data, the possibility of linking experiments and resources, and the usage of templates for standard experiments, which can additionally simplify the planning of experiments. Furthermore, most ELNs can also contribute to the simplification of lab management aspects as inventory management, sample tracking, communication, and digitalization of paperwork. Finally, in our experience data recording and documentation is done more carefully, when researchers have in mind, that their records are visible to others. ^4^

## eLabFTW has a combination of features making it a powerful tool to overcome hurdles

### What is eLabFTW?

The open-source ELN eLabFTW ( https://www.elabftw.net/) was developed to provide lab groups with a free to use ELN. The developers of eLabFTW being researchers themselves, provided a general purpose ELN with limited features, but a powerful database and search function. Making it easy to learn and use, yet very suitable for all kinds of research fields. It consists of an Experiment section, used for documenting experiments with a simple built in text editor and a Database section, which can be used to create entries for all kinds of items like plasmids, enzymes, primers, etc. by using the same text editor. Furthermore, these items can be imported automatically via comma separated value (csv) files by the admin. Subsequently, these items can directly be referenced from the experiment section. Experiments themselves also can be linked to other experiments, making it easy to keep a seamless documentation of projects and experiments. eLabFTW is built according to the open data principle, providing open-source code, as well as providing on board functions to easily share experiment and database entries with people in- and outside of eLabFTW via direct links, or export to PDF or ZIP files.

In our opinion, eLabFTW is well suited to overcome the aforementioned hurdles of ELN implementation in small preclinical labs, as well as in other environments and lead to an improvement of research data transparency and findability.

### eLabFTW is open-source software

Being an open-source software, there are no license or other costs to pay when acquiring eLabFTW. In addition, even if the development and support of the software is discontinued, the source code will always be available, preventing users to search for a new software solution. eLabFTW does not use any proprietary parts of code, so this property applies to the whole code of eLabFTW. Some other ELNs are open source but depend on pieces of proprietary third-party code. In addition, when needed, the code of eLabFTW can be adapted to individual needs, by trained IT-Staff in the organization.

### eLabFTW is easy to set up

Providing the option to use Docker Containers, a kind of virtual machine, which contains all the necessary software to run eLabFTW. Even a person with only intermediate IT knowledge, when following the official documentation ^9^ can install eLabFTW. The initial setup takes less than 30 minutes, on a Windows computer, as tested by us. The setup works on a server or even a simple computer running Linux, Windows or MacOS. That ability for local installation makes eLabFTW suitable for critical data and enables data storages independently from the availability of cloud services offered by other ELN companies. This ensures high data safety, as it prevents access to data from outsiders and protects from using services with uncompliant privacy policies. Backup of data can be set up using a simple script ^9^ and the backup files can be stored on a second hard drive, or on other machines, like a Network Associated Storage to enhance data safety.

### eLabFTW contains powerful tools for a safe documentation

eLabFTW offers a full text search engine, meaning it searches for all elements of the database, as well as in the written text of entries, making it easy to find older research data from yourself, and others. Additionally, eLabFTW contains a powerful search tool, which allow users to search for entries with specific properties. All entries in the experiment section of eLabFTW can be timestamped after RFC 3161 standard, allowing for full audit trail documentation. In addition, all revisions in any entry are tracked, so that it can be proved that no data were altered. Every item in eLabFTW is identified by a unique identifier (eLabID), which is independent from the given title. Hence, all objects can be unambiguously identified. Finally, all entries in eLabFTW can be shared via a unique link with people inside or outside the lab group, if eLabFTW is running on a computer with internet connection.

The features mentioned above are the most outstanding differences between eLabFTW and a traditional paper-based lab notebook, or other ELNs. However, eLabFTW provides some more useful features, which can be beneficial for a lab. Firstly, it offers a simple interface with just a text editor and some additional features like file upload and the integration of image files into the text. Furthermore, items in eLabFTW are marked by tags, instead of being ordered in a folder structure, and can be exported to different formats (pdf, zip, json) to be shared or stored externally. The visibility for each item can be restricted to oneself, a specific group of people or set to public. This might be an important feature to secure rights of research. Lastly, eLabFTW provides an application programming interface (API) that can be accessed with a specifically created python library. This is useful, for example, for automatic upload of raw data to eLabFTW.

## Implementation of eLabFTW in our lab

### Finding the right ELN

The implementation of an ELN was supposed to be the first step of a bigger project of introducing a lightweight quality management system in our lab. ^10^ An ELN was supposed to form the backbone of this QMS. For finding suitable software, we tested different trial versions of commercially and freely available ELNs. Our focus was on the usability rather than on the features, as we wanted to make transition to electronic documentation as easy as possible and many features offered by more expensive ELNs are not needed by our group. After initial testing, two commercial ELNs and eLabFTW came into closer selection. We got the notice that two ELNs in this selection are already hosted by our university, this being Labfolder and eLabFTW. After a two-week period during which both electronic lab books were tested in a small group, we decided to introduce eLabFTW to the whole lab. Although Labfolder has some features that eLabFTW does not have (support of including Excel and Word files, adaptable page layout), we decided to implement eLabFTW because of the much quicker loading times, open-source format and in our opinion a much easier usability. After introducing eLabFTW to every lab member, we decided about the most useful setup for our lab environment, to make it suitable for our QM-Project.

### Importing existing data

Before the setup of eLabFTW, we already started to introduce Standard Operating Procedures (SOPs) for our basic lab experiments. Using the database of eLabFTW proved to be a good way to distribute new versions of SOPs to every lab member effectively. As a first step, all existing SOPs were transferred into eLabFTW. The first new SOP described how to use eLabFTW and describes the form of documenting and time stamping of experiments, as well as linking database items. Secondly, we imported our complete database of vectors, primers, antibodies, enzymes, chemicals, consumables, and equipment, as well as cell lines into eLabFTW, together with the matching documentation as there are vector maps, material safety datasheets, primer sequences and melting temperature, antibody datasheets, restriction sites of restriction enzymes, cell line properties, handbooks of machinery, and so on. We also use the database to create project items, that all experiments belonging to that project can be linked to, to establish a timeline of experiments performed in that project. This was done using the import via csv files.

**Figure 1.**
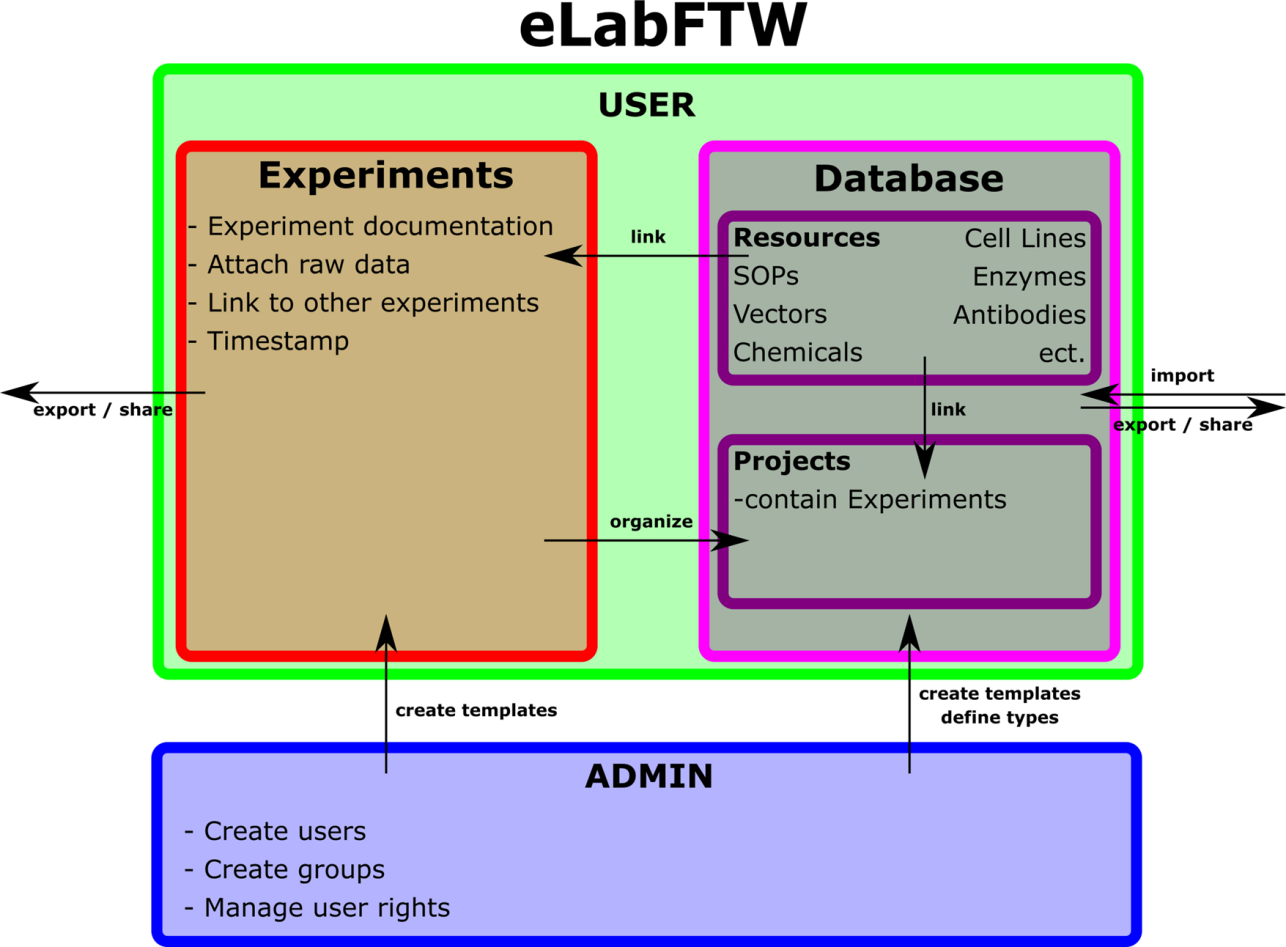
Configuration of eLabFTW as being used by our workgroup.

### Creating groups, templates, projects, and tags

We created groups of users working on specific projects, so researchers can limit the visibility of entries to the only stakeholders of that project. Some Users where put in just the general group, and some in multiple groups, depending on their projects. Templates where created for the most common experiments, performed in our lab, so researchers need less time in documenting these experiments. Also, we introduced Tags for specific experiments and Projects. Partly these are also saved in the templates of specific experiments. Tagging experiments with specific Tags works in our experience much better than saving files in specific folders, because entries can be sorted with multiple tags, in contrast to just be in one single folder. We also use the database to create project items, that all experiments belonging to that project can be linked to, to establish a timeline of experiments performed in that project. After the setup, we started using eLabFTW to upload meeting minutes and documentation of other events.

### Unused features and changes

In our lab, we do not use the booking function of lab equipment, after we found it too inconvenient after a short test period. The ELN was also supposed to be used for one-on-one project communication in form of interactive weekly progress reports, as part of our planned lightweight QMS. ^10^

This approach was ineffective, as it consumed too much time, lead to misunderstandings and was unsuitable for the discussion of some specific problems. Consequently, we switched back to personal, verbal meetings for project planning. In our experience, ELN-reporting is only suitable for one directional communication. Still this possibility of one-directional way of communication showed an unexpected advantage during the still ongoing COVID-19 pandemic. Due to electronic recording of research results and protocols, less face-to-face communication was necessary, which helped to keep the infection risk on a low level.

## Other projects dealing with ELNs

Next to our report, which focused on eLabFTW, there are also more general ELN guides from the Harvard Medical School Information Technology department 13 and the BIH14, as well as the ELN guide of the ZB Med. ^4^ Furthermore, there is the report of a research group using Evernote as their ELN. ^8^ Trend of introducing ELNs.

## Trend of introducing ELNs

Our effort embeds well in current initiatives from leading biomedical institutions, as the RE-PLACE Project at the Berlin Institute of Health (BIH), ^11^ which is an institution-wide program for adopting ELN software at the Charité and BIH, by providing free licenses to the ELN Labfolder, as well as introductory courses and support. This project is combined with an evaluation study to understand if ELN usage is indeed beneficial for fulfilling needs of researchers and to improve the quality and transparency of research documentation and data management.

The efforts of setting up eLabFTW also fit with another project our lab is involved in, the EQIPD quality system (QS). ^12^ This is the first quality system for preclinical research and eLabFTW supports our efforts for external recognition of our quality procedures and towards an EQIPD certification. Implementation of the ELN supports the implementation and maintenance of several Core Requirements (CR) set forth by the EQIPD QS. The quality system is built on 18 CR (CR1 –CR18) and eLabFTW supports the lab already with six. For example, CR12 asks that “Protocols for experimental methods must be available”, which is easily achieved with the ELN. In the same line, the CR on data records (CR6), data security (CR7), transparency (CR8) and reporting (CR9) can be easily fulfilled with such an ELN. Hence, an ELN should be considered as one of the pillars towards quality processes in a small research lab, especially when it integrates seamless in the daily lab routine as eLabFTW does.

In a forthcoming visionary standpoint, we think that stringent ELN implementation in more working units is facilitating the reduction of hurdles for interaction across disciplines, such as the interaction of basic scientists and patient-treating clinical faculty. We hypothesize this due to the fact that data can be rapidly and remotely available at any given time, not restricted to often incompatible time schedule of the participating stakeholders. Moreover, a growing body of large datasets will be summarized and presented in a more comprehensive and convenient way as compared to local storage solutions on core faculty servers or hidden folders in a given server infrastructure. Thereby, further exchanging interaction to data scientists and wet lab scientists is possible. Laboratory automation, as a standard for many work processes in industry and routine diagnostic labs, is embracing its way in many academic labs ^15^ such as in our lab occurred for a liquid handling robotics. ^16^ Direct linking of ELN to machine output, without human operator data handling step in between, saving the data file in a time stamped and logged version will minimize error introduction in parallel to increase transparency of the project.

In the future, ELNs could form the backbone of many workgroups, even small ones, to simplify research in an environment that is being more and more digitalized. This assumes that more workgroups dare to make the step to switch from traditional, paper based documentation to digital solutions. Using a software as easy to set up as eLabFTW, we think that this switch will be easy and only need small adaptations in workflow to fully use all the benefits of electronic documentation.

## Data availability

No data is associated with this article.
