# Supplementary material for: eLabFTW as an Open Science tool to improve the quality and translation of preclinical research
Source: F1000Res. 2021 Aug 2;10:292. Originally published 2021 Apr 16. [Version 3] doi: 10.12688/f1000research.52157.3 (PMC8323070; doi:10.12688/f1000research.52157.3)
Supplement: Peer review history

# Peer review: Reviewer response for version 2

**DOI:** 10.5256/f1000research.56841.r88143

**Published:** 2021-07-29

**Authors:** Amiram Goldblum

**Affiliations:** 1 Molecular Modeling Laboratory, Institute for Drug Research, The Hebrew University of Jerusalem, Jerusalem, Israel; 2 Fraunhofer Project Center for Drug discovery and Delivery, The Hebrew University of Israel, Shomre Hahar, Israel

This article presents an electronic lab note book that is intended to surpass current ELNs in several aspects that are required to overcome or to balance the  well-known disadvantages of current ELNs. Those disadvantages  are very well presented after the introduction paragraph. Although eLabFTW presents and focuses in this paper on  experimental preclinical or partially clinical labs, I believe that it could also be easily adapted and serve a theoretical and computational focused lab such as my own.

 I have a few questions-suggestions regarding **major aspects** of this paper and a few minor issues :and typos. However, I am not suggesting any revision and my comments are only for the authors to consider:

1. There seems to be a gap between the attention and interest of eLABFTW for protecting personal data versus the need to stimulate replicability of results. Internal replicability is important and seems to be a cornerstone of eLabFTW, but a major issue is replicability by other labs., and that requires communication with the "outside world".
2. The issue of communicating to the world is not specified as a component of eLabFTW: Would it support easier reporting in the form of  manuscripts, posters, talks, presentations?  Would it help in writing the more diverse "methods" and "results" sections in MS?
3. Do the search options enable advanced boolean searches?
4. Up to what size of lab would the authors suggest to use eLabFTW?

**Minor issues:** Please explain all acronyms - first-what is **FTW**?...QM (Quantum Mechanics in my world...) and QMS, RFC 3161, CR etc...each unexplained acronym reduces the reader's attention. 

**Typos:  **

- First line in "What is eLabFTW on page 3: "with a free **(ability?)** to use ELN.
- Line 3 in "Creating groups, templates etc." (page 50 should be **Templates were created)**
- Line 1 second parag under "unused features and changes" (page 6) "it consumed too much time, **led** to misunderstandings... (led is the past of lead...).
- Line 3 under "Other projects dealing with ELNs (page 6) - the last sentence "Trend of introducing ELNs" should be erased, it was probably confused with the next paragraph title. 

Is the topic of the opinion article discussed accurately in the context of the current literature?

Yes

Are arguments sufficiently supported by evidence from the published literature?

Yes

Are all factual statements correct and adequately supported by citations?

Yes

Are the conclusions drawn balanced and justified on the basis of the presented arguments?

Yes

Reviewer Expertise:

I lead a computational lab that focuses on molecular modeling and drug discovery and design.

I confirm that I have read this submission and believe that I have an appropriate level of expertise to confirm that it is of an acceptable scientific standard.

# Response

**Published:** 2021-07-29

**Authors:** Michael Hewera

**Affiliations:** Universitätsklinikum Düsseldorf, Germany

Dear Amiram Goldblum,

 I thank you a lot for reviewing our publication.

 You will be happy to see, that we corrected the typos, that you pointed out, and added explanations to the abbreviations.

 The question of boolean search operators was answered as well.

 The method of sharing data with the outside world, is already explained in the old version: "as well as providing on board functions to easily share experiment and database entries with people in- and outside of eLabFTW via direct links, or export to PDF or ZIP files".

 Thanks a lot and best regards,

 Michael Hewera

# Peer review: Reviewer response for version 2

**DOI:** 10.5256/f1000research.56841.r86284

**Published:** 2021-06-14

**Authors:** Sam Parsons

**Affiliations:** 1 Department of Experimental Psychology, University of Oxford, Oxford, UK

The revisions to the manuscript have strengthened the clarity of presentation, and streamlined the reading. It is much clearer what messages readers should take away. The restructuring is something in particular that makes this a stronger manuscript. I have a few very minor suggestions that I think would improve the manuscript.

1. In the abstract, “self-guided approach”. I may be misunderstanding – I interpret this as indicating that the manuscript will be a guide for me as a reader to implement eLabFTW. But, the paper reads as reflecting on the authors experience. Likely only a minor issue, as “In a self-guided approach” could be removed from this sentence.
2. In the first paragraph, I am unsure if replication is intended to indicate using the same data as the original paper or running the same study to collect new data with the same procedures. Reproducibility has been described as both, so it would be nice to specify.
3. A very minor point on the introduction section – I think an explicit sentence stating that this manuscript focuses on eLabFTW could streamline this section a little more.
4. Very minor – in the “eLabFTW is easy to set up” subsection: the opening sentence feels out of place and may be missing something, perhaps it belongs later in the paragraph? I was not sure if the docker aspect was part of the set up or a powerful extra.
5. A few words on the RFC 3161 standard, and why this is of benefit, would be a useful addition.
6. The QMS abbreviation is not explained, could this be laid out fully or the full title used instead?
7. A citation or website for the Labfolder software should probably be included.
8. It may be useful to also reflect on the limitations or stumbling blocks the authors found with eLabFTW. This is somewhat indicated by the ‘unused features and changes’ section, but I wonder if there are useful thoughts the authors might share on this too.

Is the topic of the opinion article discussed accurately in the context of the current literature?

Yes

Are arguments sufficiently supported by evidence from the published literature?

Yes

Are all factual statements correct and adequately supported by citations?

Partly

Are the conclusions drawn balanced and justified on the basis of the presented arguments?

Yes

Reviewer Expertise:

Openness and reproducibility, cognitive affective, psychometrics.

I confirm that I have read this submission and believe that I have an appropriate level of expertise to confirm that it is of an acceptable scientific standard.

# Peer review: Reviewer response for version 1

**DOI:** 10.5256/f1000research.55395.r84047

**Published:** 2021-05-06

**Authors:** Sam Parsons

**Affiliations:** 1 Department of Experimental Psychology, University of Oxford, Oxford, UK

The manuscript describes the open-source electronic lab notebook eLabFTW and the authors experience with implementing this into their workflow. I see this as a potentially useful contribution to the literature, in part because I am broadly positive about the use of ELNs. The majority of my comments are based around improving the clarity of the manuscript – I have numbered suggestions for ease.

1. The main point (which ties in to many of the below suggestions for clarity) I am not clear on after reading the manuscript, is the framing or the take-home message I should be left with. The content included is informative, but moves between; support or advocacy for the eLabFTW program, reflections on the groups experiences implementing it, and reflections on the good work for open science the authors groups and institutions are doing. Throughout, it feels that these aims are merged together, which makes the flow of the paper difficult to follow. I have several general suggestions that could help improve this clarity:
  1. Consider more subheadings to distinguish the message of each subsection. At a minimum, I think it would be valuable to separate or clearly distinguish sections. e.g. descriptions of eLabFTW’s technical details and instructions from the authors reflections on implementing eLabFTW. Greater use of subheadings would help the flow of the manuscript and reader clarity.
  2. Given that the abstract describes the paper as providing a self-guided approach to using eLabFTW, this section could benefit from more detail. Reading the abstract, I thought (though this may be just my reading) that this would be the core element of the paper but this section was quickly moved into more general comments on other initiatives. Further detail, and examples such as the groups own ELN, would make this a much more useful contribution.
  3. Consider removing some references to work specific to the authors groups/institutions – or, revise so that it can be made relevant to the readers context in using ELNs. For example, as a reader I did not find the closing sentence to be useful to my understanding of the eLabFTW tool “Those aspects are only a few cornerstones that shall be a focus of operations developments of a state-of-the-art translational research lab, currently underway in the lab of neurosurgery at the University Clinic Düsseldorf”. The sentences describing the good work towards openness will be maximally useful to the reader if they are situated within how they can implement these tools themselves, otherwise they are not necessary to the manuscript and distract from the main content.
2. The manuscript mentions ‘As described in one of our previous publications’ several times. My original suggestion was that more description of what was discussed would be valuable in this manuscript, else the reader is missing potentially vital information to this context. Having now read the cited paper, I am less clear of the purpose of the “Implementation of eLabFTW in our lab” section of this manuscript, which in turn leaves me less clear of the purposes of this manuscript, which related to my first point also. Perhaps the authors can more clearly differentiate between the contribution both papers make and what this manuscript adds in particular?

The following comments relate to more specific sections of the manuscript.

1. In the opening paragraph, it may be useful to differentiate between the authors definition of reproducibility and replicability. Later in the paragraph focus is placed mainly on data management, so I thought that computational reproducibility was the focus (same data + same analyses = same results) whereas the definition used was what others might describe as replicability (new data with the same procedures = same results). Not that I have a problem with the definition used, but the clarity for the focus or benefits of ELNs could be improved with this.
2. Minor typo: “unaltered toegether” I think that a word might also be missing here, perhaps ‘in an unaltered format together with…’ ?
3. “The pros and cons of using ELNs have been discussed for a long time” – Perhaps tie this in to the following two sections, or provide citations here?
4. I suggest moving around some of the text in the advantages and hurdles sections – it was slightly confusing to read as the advantages section first highlighted disadvantages and the hurdles also starts with an entire paragraph on the benefits. As mentioned above, citing examples that highlight the benefits would be valuable here too.
5. Perhaps the authors could be clearer about the focus on life sciences. Given that this is the context the ELN was implemented in, it makes sense. But, it might be useful to highlight or discuss further the use of ELNs in other contexts, or state that this paper focuses on the life sciences solely.
6. I suggest splitting the long paragraph discussing the open-source aspect of eLabFTW at least into a paragraph on open-source and a paragraph on the set up (further use of subheadings would assist here too).
7. I suggest removing the SOP acronym as it is not needed.
8. I was not sure of the purpose of the paragraph on EQIPD, it did not feel relevant to the ELN discussion.
9. Perhaps the paragraph on other guides for ELNs could be integrated to somewhere else in the manuscript?

In the interest of openness I always sign my reviews,

 Sam Parsons

Is the topic of the opinion article discussed accurately in the context of the current literature?

Yes

Are arguments sufficiently supported by evidence from the published literature?

Yes

Are all factual statements correct and adequately supported by citations?

Partly

Are the conclusions drawn balanced and justified on the basis of the presented arguments?

Yes

Reviewer Expertise:

Openness and reproducibility, cognitive affective, psychometrics.

I confirm that I have read this submission and believe that I have an appropriate level of expertise to confirm that it is of an acceptable scientific standard, however I have significant reservations, as outlined above.

# Response

**Published:** 2021-05-12

**Authors:** Michael Hewera

**Affiliations:** Universitätsklinikum Düsseldorf, Germany

Dear Dr. Parsons,

 I (in the name of my co-authors, as well) thank you for reviewing our publication and for your valuable feedback.

 We tried to implemented most of your suggestions in the new version of our publication.

 These are (not only)

 - Condensing the part about pros and cons of ELNs and sortig it

 - Specifying references to "prior publication"

 - moving text, and creating new headings/sub-headings

 - Better explain the context of eLabFTW and EQIPD 

 - Streamline the last chapter

 Best regards,

 Michael Hewera
